# Cu_2_ZnSiS_4_

**DOI:** 10.1107/S1600536811008889

**Published:** 2011-03-15

**Authors:** Kimberly A. Rosmus, Jennifer A. Aitken

**Affiliations:** a Department of Chemistry and Biochemistry, Duquesne University, 600 Forbes Avenue, Pittsburgh, PA 15282, USA

## Abstract

Single crystals of Cu_2_ZnSiS_4_, dicopper(I) zinc silicon tetrasulfide, have been prepared *via* high-temperature solid-state synthesis. Cu_2_ZnSiS_4_ was found to have the wurtz-stannite structure type, like that of Li_2_CdGeS_4_, Li_2_CdSnS_4_, and Cu_2_CdSiS_4_. Each sulfur anion is tetra­hedrally coordinated by two Cu cations, one Si cation, and one Zn cation, forming a three-dimensional honeycomb structure. When viewed along the *c* axis, the atoms are aligned in rows in which each cation alternates with the sulfur anions.

## Related literature

For synthetic procedures, see: Himmrich & Haeuseler (1991 ▶); Nitsche *et al.* (1967 ▶); Yao *et al.* (1987 ▶). For related structures, see: Chapuis & Niggli (1972 ▶); Lekse *et al.* (2008 ▶, 2009 ▶); Schäfer & Nitsche (1974 ▶). For optical properties, see: Levcenco *et al.* (2010 ▶).

## Experimental

### 

#### Crystal data

Cu_2_ZnSiS_4_
*M* *_r_* = 348.78
Orthorhombic, 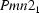

*a* = 7.4374 (1) Å
*b* = 6.4001 (1) Å
*c* = 6.1394 (1) Å
*V* = 292.24 (1) Å^3^
*Z* = 2
Mo *K*α radiation
μ = 12.77 mm^−1^
*T* = 296 K
0.13 × 0.07 × 0.06 mm

#### Data collection

Bruker SMART APEX diffractometer
Absorption correction: multi-scan (*SADABS*; Sheldrick, 2002 ▶) *T*_min_ = 0.290, *T*_max_ = 0.500
5153 measured reflections
1078 independent reflections
1023 reflections with *I* > 2σ(*I*)
*R*_int_ = 0.021

#### Refinement

*R*[*F* ^2^ > 2σ(*F* ^2^)] = 0.020
*wR*(*F* ^2^) = 0.051
*S* = 1.14
1078 reflections
44 parameters
1 restraint
Δρ_max_ = 0.72 e Å^−3^
Δρ_min_ = −1.01 e Å^−3^
Absolute structure: Flack (1983 ▶), 449 Friedel pairs
Flack parameter: 0.02 (1)

### 

Data collection: *SMART* (Bruker, 1998 ▶); cell refinement: *SAINT* (Bruker, 1998 ▶); data reduction: *SAINT*; program(s) used to solve structure: *SHELXS97* (Sheldrick, 2008 ▶); program(s) used to refine structure: *SHELXL97* (Sheldrick, 2008 ▶); molecular graphics: *CrystalMaker* (Palmer, 2010 ▶); software used to prepare material for publication: *publCIF* (Westrip, 2010 ▶).

## Supplementary Material

Crystal structure: contains datablocks I, global. DOI: 10.1107/S1600536811008889/si2337sup1.cif

Structure factors: contains datablocks I. DOI: 10.1107/S1600536811008889/si2337Isup2.hkl

Additional supplementary materials:  crystallographic information; 3D view; checkCIF report

## Figures and Tables

**Table 1:** Selected bond lengths (Å)

Cu1—S2^i^	2.3170 (7)
Cu1—S3	2.325 (1)
Cu1—S1	2.3270 (6)
Cu1—S3^ii^	2.3426 (7)
Zn1—S2	2.322 (1)
Zn1—S1	2.322 (1)
Zn1—S3^iii^	2.3650 (7)
Zn1—S3^iv^	2.3650 (7)
Si1—S1	2.131 (1)
Si1—S3^v^	2.136 (1)
Si1—S3^vi^	2.136 (1)
Si1—S2^vii^	2.143 (3)

Symmetry codes: (i) 
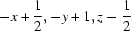
; (ii) 
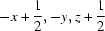
; (iii) 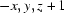; (iv) 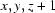; (v) 
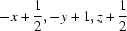
; (vi) 
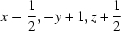
; (vii) 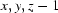.

**Fig. 1.**
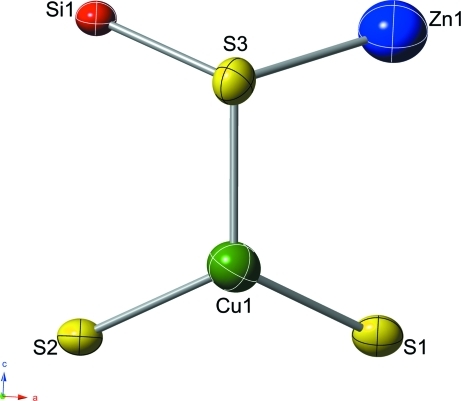
Asymetric unit of Cu2ZnSiS4 using 95% probability thermal ellipsoids.

**Fig. 2.**
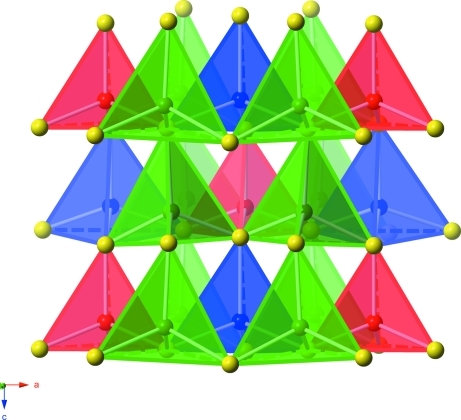
Polyhedral view down the b axis of Cu2ZnSiS4 with sulfur anions as the corners of each tetrahedron.

**Fig. 3.**
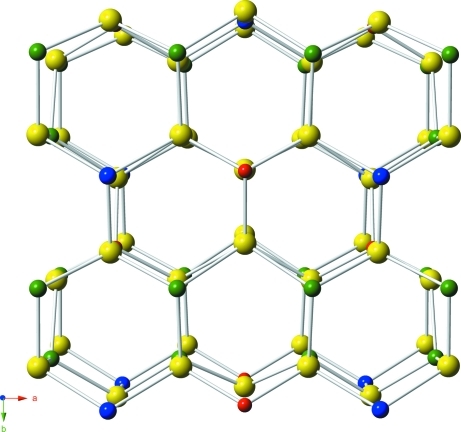
Cu2ZnSiS4 viewed along the c axis showing a three-dimensional honeycomb structure where the atoms are aligned in rows with each cation alternating with sulfur anions. Color code: Cu - green, Zn - blue, Si - red, and S - yellow.

## supplementary crystallographic information

### Comment

Cu_2_ZnSiS_4_ was prepared as crystals *via* iodine vapor transport reactions as early as 1967 (Nitsche *et al.*, 1967); however, only lattice parameters were reported. Using the same synthetic method to prepare Cu_2_ZnSiS_4_, Yao *et al.* reported the infrared spectrum of this compound (Yao *et al.*, 1987). Alternatively Cu_2_ZnSiS_4_ can be synthesized by grinding stoichiometric amounts of the elements and reacting them in a vibrational mill multiple times during the heating process (Himmrich & Haeuseler, 1991). More recently, the band gap of the title compound has been reported (Levcenco *et al.*, 2010). In this paper, Cu_2_ZnSiS_4_ was prepared as relatively small single crystals using a simple high-temperature solid-state synthesis.

Cu_2_ZnSiS_4_ possesses the wurtz-stannite structure type (Schäfer, & Nitsche, 1974) like that of Li_2_CdGeS_4_, Li_2_CdSnS_4_ (Lekse *et al.*, 2009), and Cu_2_CdSiS_4_ (Chapuis & Niggli, 1972). The asymmetric unit can be observed in Figure 1. Cu_2_ZnSiS_4_ has a diamond-like structure, where every cation is tetrahedrally coordinated with sulfur anions. The bond lengths for M—S range from 2.3170 (7)–2.3426 (7)Å for *M*=Cu, 2.322 (1)–2.3650 (7)Å for *M*=Zn, and 2.131 (1)–2.143 (3)Å for *M*=Si (Table 1). Every MS_4_ tetrahedron points in the same direction along the crystallographic *b* axis rendering the structure noncentrosymmetric (Fig.2). When viewed down the *c* axis, the ions are aligned in rows where each cation alternates with the sulfur anions (Fig.3).

Recently second harmonic generation for a couple of compounds of this structure type, Li_2_CdGeS_4_ and Li_2_CdSnS_4_, have been reported on powder samples (Lekse *et al.*, 2009). Therefore it is of interest to further study Cu_2_ZnSiS_4_.

### Experimental

Cu_2_ZnSiS_4_ was prepared *via* high-temperature solid-state synthesis. Stoichiometric ratios of the elements were weighed and then ground for 30 min in an argon-filled glovebox using an agate mortar and pestle. The sample was placed into a graphite crucible, which was then inserted in a 12 mm outer diameter fused-silica tube. The tube was flame sealed under a vacuum of 10^-3^ mbar and transported to a computer-controlled furnace. The sample was heated to 1000°C in 12hrs, held at 1000°C for 168hrs and then cooled at 7.5°C/hr to room temperature. When removed from the furnace, blue rod-like crystals of approximate size 0.13 *x* 0.07 *x* 0.6 mm were found under a light microscope.

### Crystal data

**Table:**

Cu_2_ZnSiS_4_	*F*(000) = 332
*M**_r_* = 348.78	*D*_x_ = 3.964 Mg m^−^^3^
Orthorhombic, *P**m**n*2_1_	Mo *K*α radiation, λ = 0.71073 Å
Hall symbol: P 2ac -2	Cell parameters from 3127 reflections
*a* = 7.4374 (1) Å	θ = 3.2–32.2°
*b* = 6.4001 (1) Å	µ = 12.77 mm^−^^1^
*c* = 6.1394 (1) Å	*T* = 296 K
*V* = 292.24 (1) Å^3^	Rod, blue
*Z* = 2	0.13 × 0.07 × 0.06 mm

### Data collection

**Table:**

Bruker SMART APEX diffractometer	1078 independent reflections
Radiation source: fine-focus sealed tube	1023 reflections with *I* > 2σ(*I*)
graphite	*R*_int_ = 0.021
φ and ω scans	θ_max_ = 32.9°, θ_min_ = 3.2°
Absorption correction: multi-scan (*SADABS*; Sheldrick, 2002)	*h* = −11→11
*T*_min_ = 0.290, *T*_max_ = 0.500	*k* = −9→9
5153 measured reflections	*l* = −9→9

### Refinement

**Table:**

Refinement on *F*^2^	Secondary atom site location: difference Fourier map
Least-squares matrix: full	*w* = 1/[σ^2^(*F*_o_^2^) + (0.0067*P*)^2^ + 0.2702*P*] where *P* = (*F*_o_^2^ + 2*F*_c_^2^)/3
*R*[*F*^2^ > 2σ(*F*^2^)] = 0.020	(Δ/σ)_max_ < 0.001
*wR*(*F*^2^) = 0.051	Δρ_max_ = 0.72 e Å^−^^3^
*S* = 1.14	Δρ_min_ = −1.01 e Å^−^^3^
1078 reflections	Extinction correction: *SHELXL97* (Sheldrick, 2008), Fc^*^=kFc[1+0.001xFc^2^λ^3^/sin(2θ)]^-1/4^
44 parameters	Extinction coefficient: 0.025 (1)
1 restraint	Absolute structure: Flack (1983), 449 Friedel pairs
Primary atom site location: structure-invariant direct methods	Flack parameter: 0.02 (1)

### Special details

**Table:**

Geometry. All e.s.d.'s (except the e.s.d. in the dihedral angle between two l.s. planes) are estimated using the full covariance matrix. The cell e.s.d.'s are taken into account individually in the estimation of e.s.d.'s in distances, angles and torsion angles; correlations between e.s.d.'s in cell parameters are only used when they are defined by crystal symmetry. An approximate (isotropic) treatment of cell e.s.d.'s is used for estimating e.s.d.'s involving l.s. planes.
Refinement. Refinement of *F*^2^ against ALL reflections. The weighted *R*-factor *wR* and goodness of fit *S* are based on *F*^2^, conventional *R*-factors *R* are based on *F*, with *F* set to zero for negative *F*^2^. The threshold expression of *F*^2^ > σ(*F*^2^) is used only for calculating *R*-factors(gt) *etc*. and is not relevant to the choice of reflections for refinement. *R*-factors based on *F*^2^ are statistically about twice as large as those based on *F*, and *R*- factors based on ALL data will be even larger.

### Fractional atomic coordinates and isotropic or equivalent isotropic displacement parameters (Å^2^)

**Table:**

	*x*	*y*	*z*	*U*_iso_*/*U*_eq_	
Cu1	0.24741 (3)	0.17426 (4)	0.33723 (8)	0.0133 (1)	
Zn1	0.0000	0.34747 (7)	0.84124 (15)	0.0211 (1)	
Si1	0.0000	0.6743 (1)	0.3451 (4)	0.0071 (1)	
S1	0.0000	0.3611 (1)	0.4632 (1)	0.0094 (1)	
S2	0.0000	0.6784 (1)	0.9961 (2)	0.0089 (2)	
S3	0.26269 (8)	0.1724 (1)	−0.0411 (1)	0.0100 (1)	

### Atomic displacement parameters (Å^2^)

**Table:**

	*U*^11^	*U*^22^	*U*^33^	*U*^12^	*U*^13^	*U*^23^
Cu1	0.0141 (1)	0.0135 (1)	0.0125 (2)	−0.0007 (1)	−0.0008 (1)	0.0000 (2)
Zn1	0.0235 (2)	0.0210 (2)	0.0191 (3)	0.000	0.000	−0.0016 (3)
Si1	0.0078 (3)	0.0077 (3)	0.0058 (5)	0.000	0.000	0.0007 (4)
S1	0.0126 (3)	0.0072 (3)	0.0085 (5)	0.000	0.000	0.0011 (4)
S2	0.0099 (3)	0.0104 (3)	0.0064 (6)	0.000	0.000	−0.0001 (3)
S3	0.0089 (2)	0.0101 (3)	0.0110 (5)	−0.0012 (1)	0.0006 (3)	0.0000 (3)

### Geometric parameters (Å, °)

**Table:**

Cu1—S2^i^	2.3170 (7)	Si1—S3^vi^	2.136 (1)
Cu1—S3	2.325 (1)	Si1—S2^vii^	2.143 (3)
Cu1—S1	2.3270 (6)	S1—Cu1^viii^	2.3270 (6)
Cu1—S3^ii^	2.3426 (7)	S2—Si1^iv^	2.143 (3)
Zn1—S2	2.322 (1)	S2—Cu1^vi^	2.3170 (7)
Zn1—S1	2.322 (1)	S2—Cu1^v^	2.3170 (7)
Zn1—S3^iii^	2.3650 (7)	S3—Si1^i^	2.136 (1)
Zn1—S3^iv^	2.3650 (7)	S3—Cu1^ix^	2.3426 (7)
Si1—S1	2.131 (1)	S3—Zn1^vii^	2.3650 (7)
Si1—S3^v^	2.136 (1)		
			
S2^i^—Cu1—S3	112.51 (4)	Si1—S1—Zn1	112.05 (8)
S2^i^—Cu1—S1	106.98 (3)	Si1—S1—Cu1^viii^	111.72 (5)
S3—Cu1—S1	111.92 (4)	Zn1—S1—Cu1^viii^	108.24 (4)
S2^i^—Cu1—S3^ii^	106.09 (4)	Si1—S1—Cu1	111.72 (5)
S3—Cu1—S3^ii^	108.38 (3)	Zn1—S1—Cu1	108.24 (4)
S1—Cu1—S3^ii^	110.82 (4)	Cu1^viii^—S1—Cu1	104.51 (4)
S2—Zn1—S1	112.01 (5)	Si1^iv^—S2—Cu1^vi^	115.21 (4)
S2—Zn1—S3^iii^	107.88 (4)	Si1^iv^—S2—Cu1^v^	115.21 (4)
S1—Zn1—S3^iii^	108.84 (4)	Cu1^vi^—S2—Cu1^v^	108.34 (5)
S2—Zn1—S3^iv^	107.88 (4)	Si1^iv^—S2—Zn1	113.46 (6)
S1—Zn1—S3^iv^	108.84 (4)	Cu1^vi^—S2—Zn1	101.47 (4)
S3^iii^—Zn1—S3^iv^	111.40 (5)	Cu1^v^—S2—Zn1	101.47 (4)
S1—Si1—S3^v^	108.68 (7)	Si1^i^—S3—Cu1	111.38 (7)
S1—Si1—S3^vi^	108.68 (7)	Si1^i^—S3—Cu1^ix^	110.92 (5)
S3^v^—Si1—S3^vi^	111.40 (7)	Cu1—S3—Cu1^ix^	108.76 (3)
S1—Si1—S2^vii^	110.60 (9)	Si1^i^—S3—Zn1^vii^	111.42 (5)
S3^v^—Si1—S2^vii^	108.74 (7)	Cu1—S3—Zn1^vii^	105.21 (4)
S3^vi^—Si1—S2^vii^	108.74 (7)	Cu1^ix^—S3—Zn1^vii^	108.95 (3)

Symmetry codes: (i) −*x*+1/2, −*y*+1, *z*−1/2; (ii) −*x*+1/2, −*y*, *z*+1/2; (iii) −*x*, *y*, *z*+1; (iv) *x*, *y*, *z*+1; (v) −*x*+1/2, −*y*+1, *z*+1/2; (vi) *x*−1/2, −*y*+1, *z*+1/2; (vii) *x*, *y*, *z*−1; (viii) −*x*, *y*, *z*; (ix) −*x*+1/2, −*y*, *z*−1/2.
